# The Premalignant Ancestor Cell of t(14;18)^+^ Lymphoma

**DOI:** 10.1097/HS9.0000000000000579

**Published:** 2021-06-01

**Authors:** Gabriel Brisou, Bertrand Nadel, Sandrine Roulland

**Affiliations:** 1 Hematology Department, Institut Paoli-Calmettes, Marseille, France; 2 Aix Marseille University, CNRS, INSERM, CIML, Marseille, France

## Follicular lymphoma development

Follicular lymphoma (FL) is the second most common adult lymphoma and, with an incidence of ~4/100,000/year, represents around a fourth of non-Hodgkin lymphoma new diagnoses.^1,2^ The clinical course is typically indolent, and thanks to great advances in patient management with the introduction of immunochemotherapy in the 2000s, median overall survival now reaches 10 to 14 years.^3^ Yet, 2 large problems persist. First, a fraction of patients (~20%) will show early progression (before 24 mo) and/or transformation into high-grade lymphoma with poor prognosis.^4^ For the remaining 80% of patients, the clinical course is rhythmed by recurrent, shorter, and increasingly chemoresistant relapses.^3^ Most patients are diagnosed at an advanced stage (bone marrow [BM] involvement in ~40% cases, up to 70% with sensitive polymerase chain reaction [PCR]), and altogether FL is still considered incurable with available treatments.^3^ A number of tools have been developed to predict high-risk FL. Progression of disease within 24 months (POD24) is a reference posttreatment marker, but not designed to guide upfront treatment.^4^ FLIPI and FLIPI2 are important predictors of outcome, but display low sensitivity to predict POD24.^5,6^ The design of m7-FLIPI, PRIMA-PI, and 23-gene scores improved sensitivity, but their prognostic values remain largely dependent of the therapeutic regimens.^7–10^ Altogether, the overall accuracy of predictor scores and the identification of potential therapeutic targets are fundamentally hindered by high intratumoral subclonal complexity in FL, which is intrinsically linked to FL etiology.^11,12^ The 2 major current clinical challenges are thus: (1) to optimize treatment at diagnosis, especially through identifying the 20% high-risk patients and providing adapted (targeted?) therapy and (2) to address the issue of chemoresistant relapses. This requires further defining the biology of the clones from which relapses emerge, and detailed understanding of the steps underlying clonal progression.

Why is FL irremediably relapsing? Part of the answer comes from FL’s indolent clonal evolution. The disease emergence and subsequent course is a long (indolent) process, starting years—if not decades—before diagnosis. Overt FL is indeed preceded by an insidious phase of asymptomatic growth, likely emerging from widely disseminated precursor clones evolving over time.^13–17^ Genetic studies of paired diagnosis/relapse FL samples have shown that relapses rarely derive from direct evolution of the dominant clone at diagnosis, rather, arise from divergent evolution of antecedent, less evolved ancestral clones.^18–21^ This suggests the existence of a precursor population before disease onset, which serves as a root to propagate relapse. That such precursors can be branched at various stages of FL development (including early and nontransformed) and seed relapses is a fundamental paradigm shift in the FL genesis model with a major clinical impact. The occurrence of recurrent relapses following remission to immunochemotherapy in the vast majority of patients indicates that such regimens likely do not eradicate the FL-committed precursor subclones. Addressing the relapse issue is thus intimately linked to the eradication of such committed precursors that evade therapy for an unknown reason, and will not be achieved with therapies targeting late mutational events present only in evolved FL subclones. Characterizing and mapping the genetic precursor mutational landscape is a new and major challenge currently ongoing in several research laboratories.

One of the main problems in addressing the committed precursor clones (CPC) is that FL follows a complex evolution over many decades in asymptomatic individuals, which eventually results in a large interpatient and intrapatient spatiotemporal tumor heterogeneity representing a major hurdle to current therapy.

**Figure 1.**
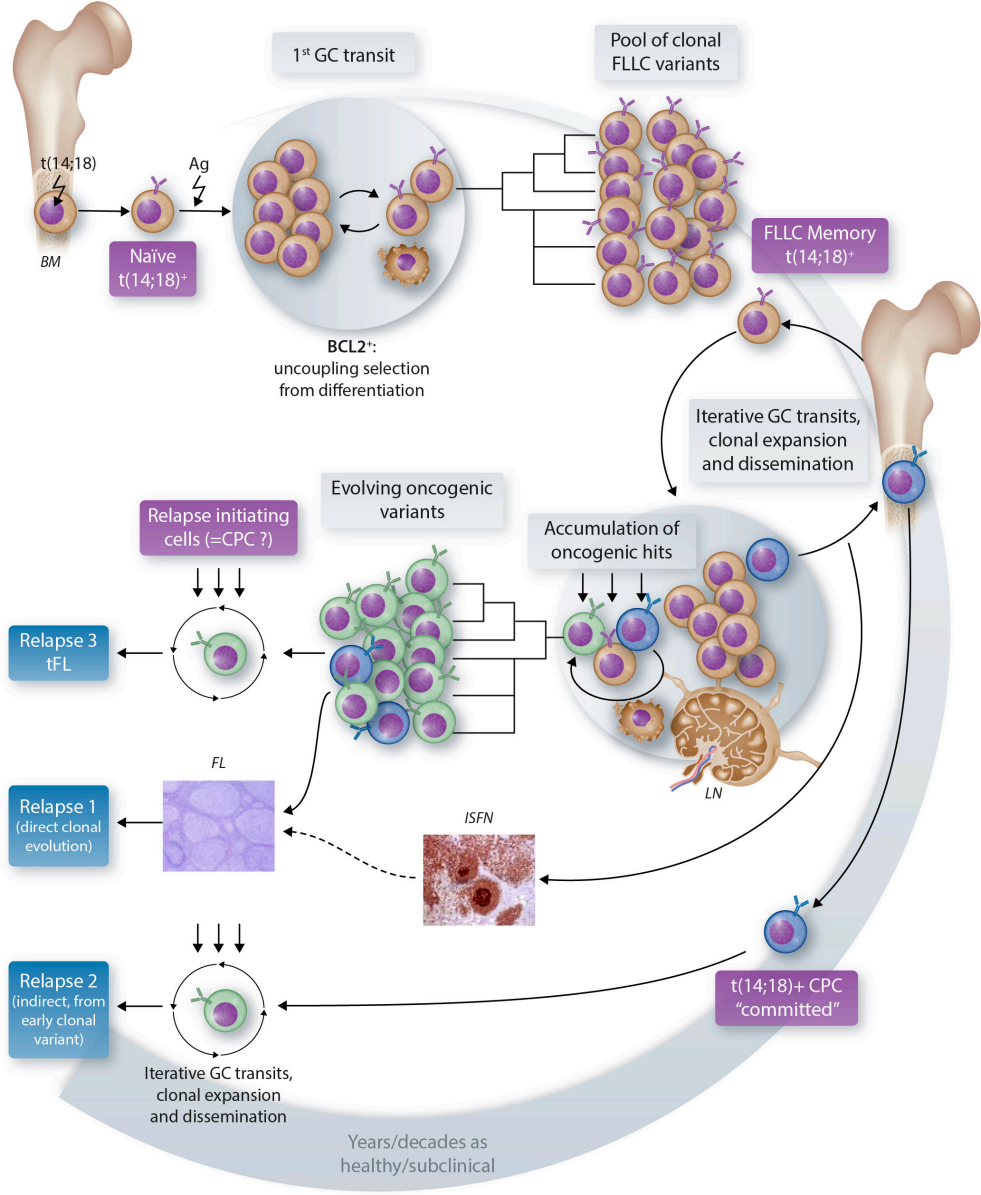
**The current model of FL development.** Follicular lymphoma biological complexity is depicted as resulting from a multihit pathway escalating along B cell differentiation stages over years/decades. Several premalignant intermediates have been identified or inferred as “precursors” or “CPC,” each of which could be at the origin of relapses. Characterizing such different CPC flavors (molecular/phenotypic/functional) is a current major challenge in the field. The arrows depict the supposed trajectory of follicular lymphoma precursor cells along the normal B cell differentiation path. BM = bone marrow; CPC = committed precursor clones; GC = germinal center; FL = follicular lymphoma; FLLC = follicular lymphoma like cells; ISFN = in situ follicular neoplasia; LN = lymph node; tFL = transformed follicular lymphoma.

From the biological standpoint, FL is a germinal center (GC) B cell lymphoma, which sustains multiple somatic mutations in oncogenes and tumor suppressors and thereby combines high dependency to the microenvironment, epigenetic, and transcriptomic reprogramming.^22^ Yet, the first steps of lymphomagenesis are not initiated in the GC but in the BM through the t(14;18) BCL2/IGH translocation, present in ~85% of FL cases.^23^ t(14;18) is necessary but not sufficient for malignant transformation; the best evidence being that it is found at low frequency (~1 cell per million B cells) in the peripheral blood of >70% healthy individuals, the vast majority of whom will never develop disease.^13–16^ It is now clear that FL lymphomagenesis is a multihit pathway escalating along B cell differentiation stages. Because hits are not happening all at once, this implies the presence of early FL precursors in “asymptomatic carriers,” blurring the notion of “healthy individuals” and “subclinical patients.” In the current model of FL lymphomagenesis (Figure 1), GCs stand at the heart of the generation of precursor clones and their evolution to overt FL. Indeed, while BCL2 constitutive expression allows pre B to naive B cell differentiation and BM exit as naive mature B cells, chronic (antigenic?) stimulation of such t(14;18)^+^ naive B cells will provoke iterative GC entries, generating multiple rounds of clonal expansion and antibody diversification process, and thereby increasing the risk to accumulate secondary oncogenic event.^22^ GC-experienced t(14;18)^+^ cells disseminate very early on within the whole body, including in the BM, to become advanced precursors. Because some of those premalignant intermediates could be at the origin of relapses, a better characterization of FL precursor clones is instrumental. Early FL pathogenesis is less clear in t(14;18) negative cases (15% of stage III/IV FL and 50% of stage I/II FL); however, typical FL mutations also affect t(14;18) negative FLs suggesting partially overlapping molecular pathogenesis with specific enrichment of immune-response and N-glycosylation signatures in t(14;18)-negative FL.^24^

## Committed precursor clones

One of the most remarkable direct demonstrations of the existence, in asymptomatic individuals, of a reservoir of precursors “committed” to FL development came from reports and molecular description of donor/recipient pairs who synchronously developed FL up to 9 years after BM transplantation.^25,26^ Both tumors and prediagnostic blood harbored the same malignant FL clone (same translocation breakpoint, same B cell receptor rearrangement), univocally demonstrating transfer from donor to recipient. The development of the same t(14;18)^+^ clone in 2 different hosts provided direct proof that a committed FL precursor clone can be present in BM long before diagnosis. Most importantly, deep-sequencing analysis of tumors and blood showed in 1 case the presence of 14 shared mutations (some of which were recurrent FL mutations), demonstrating that such committed precursors may have already acquired “second hits” long before clinically manifest disease.

Thus, such BM transplantation reports revealed that in rare cases, healthy individuals could carry CPCs engaged in FL development. The investigation of a large cohort of healthy individuals was then required to assess how to discriminate “at risk” individuals carrying such CPC from most individuals carrying GC-experienced t(14;18)^+^ B cells, but who will likely never develop the disease. In collaboration with epidemiologists at the International Agency for Research on Cancer (IARC), we took advantage of the large European Prospective Investigation into Cancer and Nutrition (EPIC) population-based cohort, which gathered >1/2 million healthy volunteers at enrolment, collected a library of blood DNA at time of inclusion, and followed-up for over 20 years for the onset of cancer. Among those, prediagnostic samples were compared between 165 individuals who developed FL up to 20 years later and 346 matched controls who did not develop FL in that time-frame. Blood DNA was screened qualitatively and quantitatively for t(14;18) translocation.^15^ In all cases with paired prediagnostic blood and tumor biopsy (harvested up to 10 y later), we and others could confirm an identical t(14;18) translocation, demonstrating that the progression occurred from t(14;18) precursors circulating in blood up to 10 years before diagnosis.^15,27^ Moreover, in 20% of healthy individuals who developed FL later on, pre-FL blood samples showed circulating t(14;18) frequencies above a threshold 10^−4^, significantly above the median frequency in healthy individuals. It thus seems that in a fraction of individuals, commitment to FL development is associated with proliferation and release of precursor clones from tissue to blood, and likely to BM niches, since we showed that high t(14;18) frequencies in blood correlate with high t(14;18) frequencies in the BM.

## Remaining questions and ongoing approaches to further characterize CPCs

What does it take for a t(14;18)^+^ cell to become a committed precursor? Does it consist of the accumulation of cell intrinsic genetic events, such as the acquisition of second hits, notably leading to frequent epigenetic program modifications? Or cell extrinsic signals from a remodeled tumor microenvironment promoting functional changes, such as transition of functional states due to differentiation/dedifferentiation?

The relatively high frequency of t(14;18)^+^ cells in prediagnostic blood from incident FL patients years before diagnosis provides a challenging yet unique opportunity to directly interrogate the CPC genomic landscape through ultra-deep sequencing approaches. Ongoing analysis using CAncer Personalized Profiling by deep Sequencing (CAPP-Seq) suggests a major functional role for histone acetyl transferase loss-of-function in committing t(14;18)^+^ cells in early lymphomagenesis.^28^ Accordingly, previous phylogenetic evolution studies across sequential biopsies of FL diagnosis/relapse cases implicates chromatin-modifying gene mutations as early mutational events during tumor evolution.^18–21^

### What are the functional consequences of CPC mutations and how do such mutations cooperate to progressively build heterogeneous FL tumors?

Tissue samples from various intermediate stages of FL development are only sporadically accessible in humans. Nevertheless, various mouse models have shown that CREBBP or KMT2D loss of function combined with BCL2 overexpression accelerate lymphomagenesis by altering the epigenetic control of the GC transcriptional program and decreasing the antitumor immune response.^29–34^ To investigate how epigenetic alterations shape the kinetics of FL development, we engineered a KMT2D^ko^ BCL2^+^ mouse lymphoma model that accurately mimics human lymphoma intermediates from premalignant lesions to more advanced FL-like tumors with long latencies.^35^ In biopsies from such intermediate stages, we profiled temporal changes of B cell states and investigated functional dynamics along FL tumorigenesis.

**Figure 2.**
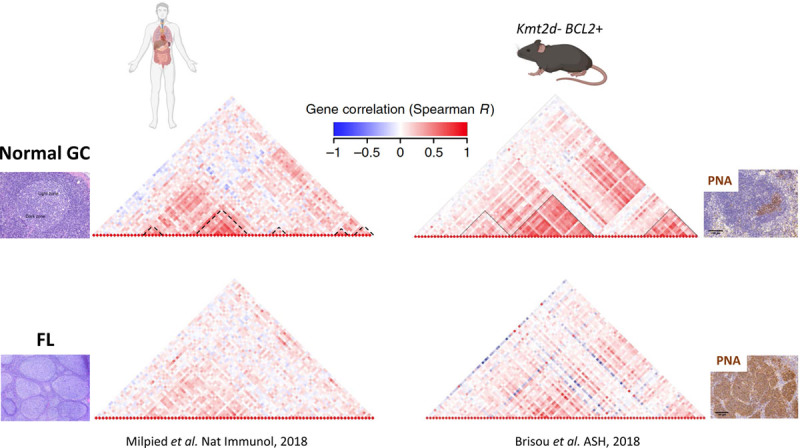
**FL malignant progression is characterized by a progressive desynchronization of a highly coordinated transcriptional program.** Dendrograms represent gene correlation computed on single cell quantitative PCR data obtained from normal GC B cells (TOP) and FL cells (DOWN) in human and in a mouse model mimicking loss of *Kmt2d* and *BCL2* overexpression (*Kmt2d*− BCL2+). The hierarchical clustering is based on gene evolution along the θ GC pseudotime, inferred from the data of thousands of single GC B cells, that model the GC cycle, allowing the identification of clusters of correlated and co-evolving genes characterizing the normal GC reaction; dashed lines at bottom of dendrograms obtained from normal GC B cells demarcate clusters of co-evolving genes during GC reaction.

Using single cell transcriptomics and pseudotime analysis in human biopsies, we were able to reconstruct the dynamics of normal GC reaction into a cyclic continuum of intermediate stages between light zone (LZ) and dark zone (DZ) B cells. We found that in physiological GC reactions, each defined intermediate stage was characterized by tightly coordinated clusters of genes whose expression co-evolved during the GC cycle. We could build a reference map of synchronized up and downregulation accompanying LZ to DZ transitions and back. Strikingly, we found that this gene expression dynamic was entirely lost in human FL.^12^ Applying the same methodology to our mouse models, pseudotime analysis of the GC trajectory showed that mouse FL tumors presented a loss of synchrony of the GC program remarkably similar to the situation observed in humans FL (Figure 2).^35^ We are currently investigating through scRNA-seq analysis the kinetics of gene expression changes in KMT2D^ko^ BCL2^+^ B cells along pre-FL to FL development. Altogether, this series of analysis should contribute to deciphering when and how the combination of BCL2 overexpression and KMT2D loss impedes normal GC reaction dynamics and contribute to the transcriptional intratumor heterogeneity observed in humans.

Altogether, the identification of mutations driving CPC commitment and the functional analysis of when and how their combined effects shape the (re)emergence of FL should help rationalize therapeutic approaches to prevent/delay relapses in the patients carrying such alterations at diagnosis.

### Therapeutic perspectives/clinical implications

Despite the fact that rituximab maintenance yields longer remissions, possibly by limiting the pool of FL precursors or their descendant subclones, we lack a detailed characterization of CPC/relapse-initiating cells allowing to propose a rationale targeted approach to eradicate this population and prevent relapse. However, the presence of chromatin modifying gene (CMG) mutations in early precursors suggests that targeting CMG mutations might be interesting for that purpose.^20,21,28,36,37^ Interestingly, CREBBP mutations and EZH2 mutations favor immune evasion through major histocompatibility complex (MHC) downregulation that has been associated with a decreased T cell infiltration. It has been shown in murine models that HDAC3 inhibitors and EZH2 inhibitors can restore MHC expression.^31,38^ Furthermore, EZH2 mutations induce a remodeling of GC environment by reprogramming how GC B cells interact with T follicular helper cells and follicular dendritic cells, creating a premalignant lymphoma niche.^39^ The EZH2 inhibitor tazemetostat produced an overall response rate of 69% (95% CI, 53-82; 31 of 45 patients) in relapsed/refractory FL patients with *EZH2* mutations and 35% (95% CI, 23-49; 19 of 54 patients) in *EZH2*^*WT*^ patients with a median duration of response of 11 months (95% CI, 7.2-not estimable) and 13 months (95% CI, 6-not estimable), respectively.^40^ The good tolerability profile of tazemetostat and its potential immunomodulating properties on MHC expression and B-T crosstalk^41^ makes it a promising candidate for combinations with immunotherapeutic approaches. Altogether this data suggest that combination of epigenetic drugs with targeted immunotherapies might constitute a good approach to eradicate FL precursors. Future trials will need to integrate novel tools to monitor FL precursors and relapse-initiating cells in blood and BM (eg, ctDNA monitoring, scRNAseq).

## Disclosures

The authors have no conflicts of interest to disclose.
